# Gelation in Photoinduced ATRP with Tuned Dispersity of the Primary Chains

**DOI:** 10.1021/acs.macromol.2c02159

**Published:** 2023-02-22

**Authors:** Frances Dawson, Hugo Jafari, Vytenis Rimkevicius, Maciej Kopeć

**Affiliations:** Department of Chemistry, University of Bath, Claverton Down, Bath BA2 7AY, U.K.

## Abstract

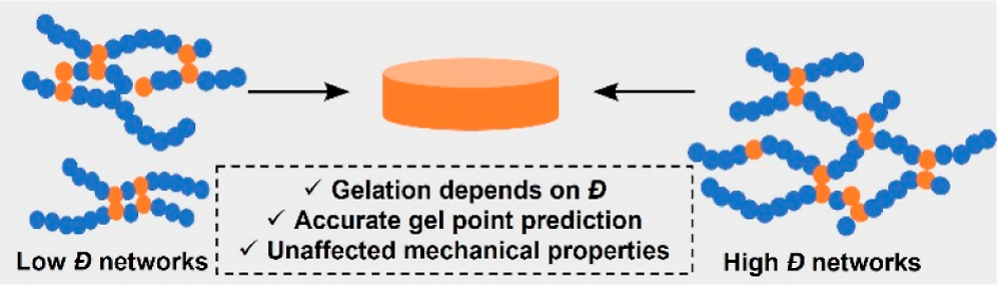

We investigated gelation in photoinduced atom transfer radical polymerization (ATRP) as a function of Cu catalyst loading and thus primary chain dispersity. Using parallel polymerizations of methyl acrylate with and without the addition of a divinyl crosslinker (1,6-hexanediol diacrylate), the approximate values of molecular weights and dispersities of the primary chains at incipient gelation were obtained. In accordance with the Flory–Stockmayer theory, experimental gelation occurred at gradually lower conversions when the dispersity of the primary chains increased while maintaining a constant monomer/initiator/crosslinker ratio. Theoretical gel points were then calculated using the measured experimental values of dispersity and initiation efficiency. An empirical modification to the Flory–Stockmayer equation for ATRP was implemented, resulting in more accurate predictions of the gel point. Increasing the dispersity of the primary chains was found not to affect the distance between the theoretical and experimental gel points and hence the extent of intramolecular cyclization. Furthermore, the mechanical properties of the networks, such as equilibrium swelling ratio and shear storage modulus showed little variation with catalyst loading and depended primarily on the crosslinking density.

## Introduction

Crosslinked polymer networks, such as thermosets, elastomers, or (hydro)gels are materials of crucial industrial importance. One of the main ways to synthesize polymer networks is free radical polymerization (FRP) of vinyl monomers with small amounts of divinyl crosslinkers; such a reaction leads to highly branched chains which eventually form an infinite network at the so-called “gel point”.^1−3^

Crosslinking in FRP can be described by the classical Flory–Stockmayer (FS) mean-field theory^3−5^ which predicts that at the critical moment of gelation,
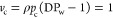
1where *v*_c_ is the weight-average number of crosslinks per primary chain (equal to 1 at incipient gelation), ρ is the fraction of the double bonds residing on the divinyl crosslinker, *p*_c_ is the conversion of the double bonds, and DP_w_ is the weight-average degree of polymerization of the primary chains in the absence of crosslinks. In FRP, polymers with high molecular weights (MWs) form immediately in the reaction; hence, gelation typically occurs at low conversions. However, the experimental gel points are still 1–2 orders of magnitude higher than those predicted by the FS theory; this is mainly caused by the intramolecular crosslinking and cyclization reactions, not accounted for by the theory,^2,3,5^ which result in the formation of local microgels and spatially inhomogeneous networks.^6,7^

Among countless polymer architectures enabled by the development of reversible deactivation radical polymerization (RDRP), these techniques have also introduced a fundamentally different mechanism of gelation.^3,8,9^ In RDRP, fast initiation and linear growth of uniform chains result in a greatly delayed (or even avoided) gelation and slow formation of branched polymer chains, leading to more homogeneous networks than in FRP. Indeed, experimental gel points in RDRP are observed at higher monomer conversions as well as closer to the theoretical values than in FRP due to less intramolecular cyclization and no microgelation.^7−9^ This effect is universal regardless of the activation/deactivation mechanism and was observed in nitroxide-mediated polymerization,^10,11^ atom transfer radical polymerization (ATRP),^12−16^ and reversible addition-fragmentation transfer (RAFT) polymerization.^17−19^

By applying the FS theory to RDRP systems, Gao and Matyjaszewski derived an expression to calculate theoretical gel points (see the Supporting Information for derivation)^8,16^
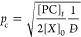
2where *p*_c_ is the conversion of vinyl bonds at incipient gelation, [PC]_*t*_ is the instantaneous concentration of primary chains at the gel point, [*X*]_0_ is the initial concentration of the crosslinker, and *D̵* is the dispersity of the primary chains in the absence of crosslinks. Furthermore, [PC]_*t*_ can be approximated as [*I*]_0_ × IE_*t*_, where [*I*]_0_ is the initial concentration of the alkyl halide initiator in ATRP (or the chain transfer agent in RAFT), and IE_*t*_ is the initiation efficiency (i.e., *M*_*n*,theo_/*M*_*n*,exp_) at the gel point.

From eq 2, gelation in ATRP depends on the initial ratio of the initiator and crosslinker and on *D̵* of the primary chains at the gel point. Interestingly, *D̵* in ATRP can be tuned by changing the catalyst loading in activator regeneration ATRP methods such as activator regenerated by electron transfer (ARGET)^20−23^ or photoinduced ATRP.^24,25^ Indeed, Li et al. have previously reported that decreasing the catalyst loading in ARGET ATRP of methyl acrylate (MA) with a crosslinker led to earlier gelation due to the higher *D̵* of the primary chains, however without comparing theoretical and experimental gel points.^26^

Notably, the difference between the theoretical and experimental gel points can be an important and useful parameter. This is because the intramolecular cyclization events, predominantly responsible for this discrepancy, result in topological defects (loops) in the network structure, which have a detrimental effect on mechanical properties such as swelling and rubber-like elasticity.^27^ While some strategies to determine and even limit loop formation in step-growth polymerization and vulcanized networks have been developed,^28−31^ similar approaches to quantify cyclization in chain-growth polymerization remain elusive. So far, the only method was proposed by Rosselgong and Armes who used ^1^H and ^13^C NMR to quantify the extent of intramolecular cyclization in branched PMMA copolymerized with a disulfide dimethacrylate crosslinker by RAFT.^32,33^ However, this method can be only employed for soluble branched polymers (i.e., pregelation) containing disulfide groups.

**Scheme 1:**
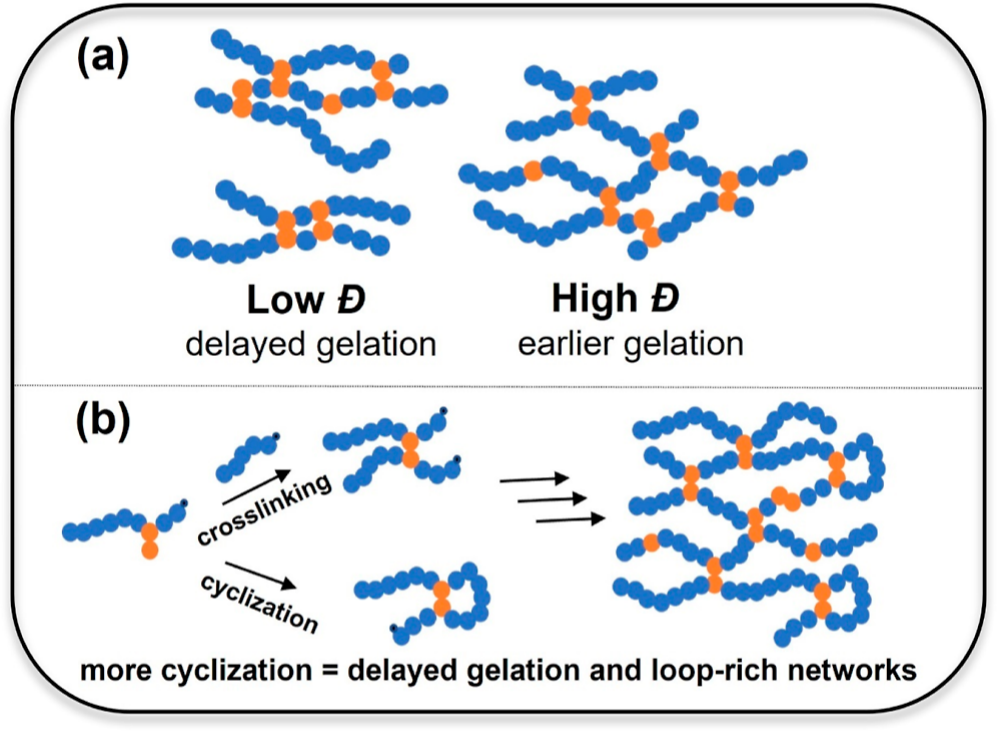
Illustration of the Effect of (a) Primary Chain Dispersity and (b) Intramolecular Cyclization on Gelation and Network Formation in ATRP

Gel point analysis can thus serve as an indirect way to estimate the loop content in gels/networks, but it requires careful calculation and comparison of the experimental and theoretical values. Notably, the effect of primary chain dispersity must be considered as higher *D̵* leads to earlier gelation (Scheme 1). Unfortunately, theoretical gel points calculated by eq 2 are usually underestimated due to the long-debated inapplicability of the FS theory to predict gelation in RDRP. More accurate gel points were obtained from computer simulation methods such as kinetic modeling or Monte Carlo and found to be significantly higher and closer to experiments than those calculated by the FS theory.^12,15,34−37^

Additionally, reports on the structural characterization of networks prepared by RDRP techniques have only recently started to emerge. For example, Appel et al. proposed a gel point normalization method in order to account for crosslinks lost due to intramolecular cyclization and rationalize the synthesis of branched/network architectures by RAFT.^38^ Konkolewicz and Matyjaszewski employed degradable crosslinkers to compare the structure and mechanical properties of networks prepared by ATRP and RAFT.^39,40^ However, an interdependence between gelation kinetics, primary chain dispersity, and physical properties of networks from RDRP has not yet been studied.

**Scheme 2:**
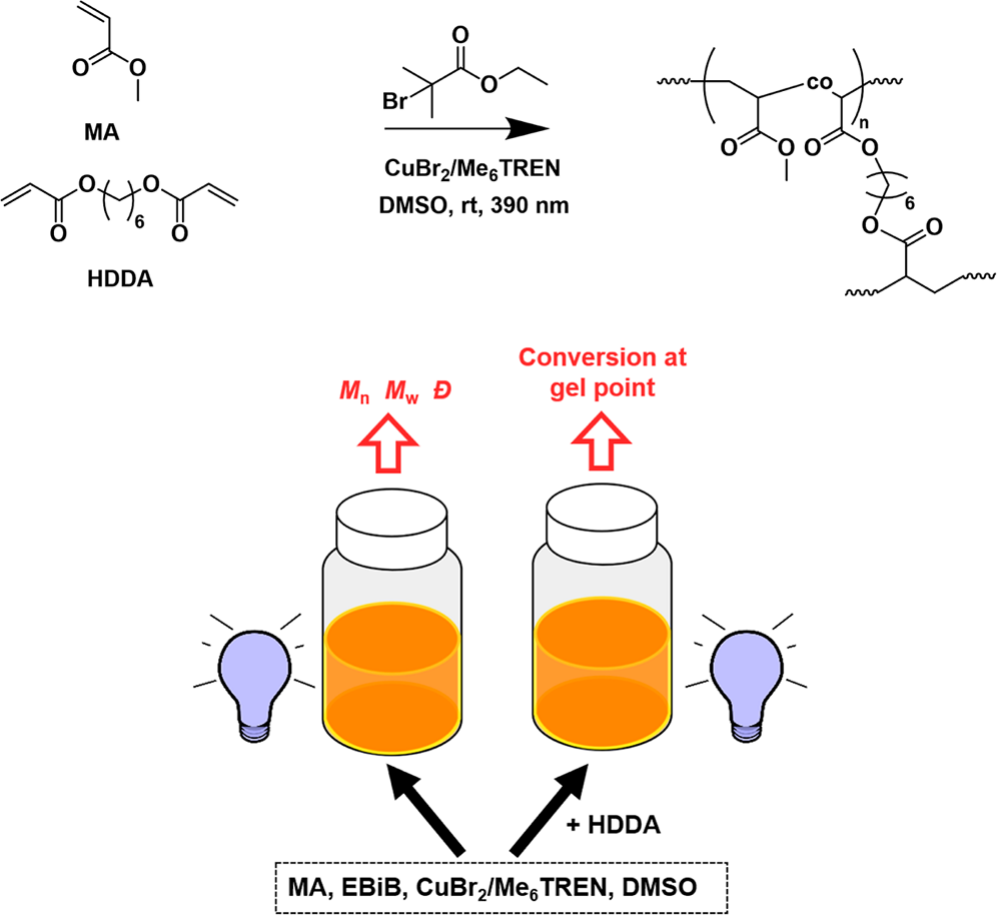
Overall Reaction Scheme and Illustration of the Experimental Setup for Parallel Photoinduced ATRP of MA with and without a Crosslinker

In this work, we investigate the effect of catalyst loading, and thus primary chain dispersity, on both experimental and theoretical gel points as well as on the mechanical properties of poly(methyl acrylate) (PMA) networks prepared by ATRP. A parallel reaction setup was used where two identical polymerizations are conducted simultaneously, with the only difference being the addition of a crosslinker (Scheme 2). In this way, the gel point conversion as well as MW and *D̵* of the primary chains could be measured at once (assuming that the primary chains in the networks are comparable with linear polymers obtained under identical conditions). Photoinduced ATRP of MA in dimethyl sulfoxide (DMSO) with 1,6-hexanediol diacrylate (HDDA) as a crosslinker was chosen as a model reaction due to the possibility of quickly turning both reactions off immediately after gelation. Moreover, photoinduced ATRP allows facile tuning of dispersity by changing the Cu catalyst loading.^24,25^

## Experimental Part

### Materials

MA (99%, Alfa Aesar), ethyl α-bromoisobutyrate (EBiB, 98%, Alfa Aesar), tris 2-(dimethylamino)ethyl amine (Me_6_TREN, 98%, Alfa Aesar), copper(II) bromide (CuBr_2_, 99%, Alfa Aesar), HDDA (99%, Alfa Aesar), DMSO (Fischer), and dimethylformamide (DMF, Fischer) were used as received. MA and HDDA were passed through a column of basic alumina to remove the inhibitor before polymerization.

### Gel Point Determination in Photoinduced ATRP of MA

A 20 mL sample vial was charged with 10 mL of MA (110 mmol, 100 equiv), 10 mL of DMSO, 0.163 mL (1.11 mmol, 1 equiv) of EBiB, and an appropriate amount of CuBr_2_/Me_6_TREN stock solution in DMSO to obtain the desired catalyst loading. A few drops of DMF were added as an internal NMR standard. The contents of the sample vial were then divided by half into two separate vials, and an appropriate amount of HDDA was added to one of the vials, namely, either 0.373 mL (1.67 mmol, 3 equiv) or 0.187 mL (0.832 mmol, 1.5 equiv). Both vials were fitted with a rubber seal and degassed with nitrogen for 15 min. After the initial sample was taken, each vial was irradiated by a Kessil PR106L-390 LED lamp (52 W, λ_max_ = 390 nm, set to 100% intensity). Samples were taken at time intervals for ^1^H NMR and gel permeation chromatography (GPC) measurements to determine monomer conversion in both reactions and the MW and dispersity of the primary chains (non-crosslinking reaction). The reactions were conducted until a gel was observed in the polymerization with a crosslinker, defined as the moment when the reaction mixture lost its mobility upon vial inversion. Both reactions were then turned off, and a final sample was taken from the nongelled reaction.

### Preparation of Fully Developed PMA Networks

A 20 mL sample vial was charged with 7 mL of MA (77.7 mmol, 100 equiv), 7 mL of DMSO, and 0.114 mL of EBiB (0.78 mmol, 1 equiv.) and stirred for 10 min 3 mL portions of this mixture were added to three vials with no screw top to make triplicate gel samples for each composition. An appropriate amount of HDDA was added to each vial, either 0.056 mL (0.25 mmol, 1.5 equiv) or 0.112 mL (0.5 mmol, 3 equiv). A few drops of DMF were added to the remaining 5 mL of MA solution to allow the reaction to be monitored for conversion by NMR. The appropriate amount of CuBr_2_/Me6TREN stock solution was added to the vials to reach the desired catalyst loading. The vials were fitted with a rubber seal and degassed with nitrogen for 30 min. The samples were irradiated by a Kessil PR106L-390 LED lamp (52 W, λ_max_ = 390 nm, set to 50% intensity) for 10 h. The gels were removed from the vials and immediately washed and dried.

### Swelling Analysis

The whole gel disks were washed in 4 × 15 mL of acetone, leaving each washing cycle overnight to ensure that all sol fraction was removed. After the final washing cycle, the mass of the swollen gel disk was recorded as *m*_swollen_. The disk was then dried in air overnight and then in a vacuum oven for 24 h. The dry gel disk mass was recorded as *m*_dry_. The equilibrium swelling ratio (ESR) was calculated as ESR = *m*_swollen_/*m*_dry_. The measurements were performed in triplicate.

### Instrumentation

^1^H NMR (Bruker AVANCE 400 MHz) was used to determine monomer conversions in CDCl_3_ using DMF as an internal reference. GPC measurements were performed on Agilent 1260 Infinity fitted with an autosampler, dual-angle light scattering system, viscometer, and refractometer, with 2 × PL gel 5 μM Mixed D columns and a guard column, with THF as the mobile phase (kinetic samples) or with 2 × PolarGel-M 8 μM columns and DMF as the mobile phase (fully developed networks pregelation). MWs were calculated using linear poly(methyl methacrylate) standards. Oscillatory rheology measurements were carried out using a TA Instruments Discovery HR-3 rheometer fitted with a 20 mm crosshatched parallel-plate geometry and a crosshatched base plate. Dry disk-shaped network samples with a thickness of 2.5 mm were assessed under a constant axial force of 1.5 N. Frequency sweeps were carried out at 25 °C over a range of 0.01–10 rad^–1^ at a constant strain of 1%.

## Results and Discussion

### Experimental Gel Points

Photoinduced ATRP of MA in DMSO with the Me_6_TREN catalyst was conducted at two different [EBiB]/[HDDA] ratios, namely, 1:3 and 1:1.5, and five different catalyst loadings, namely, 200, 100, 50, 20, and 10 ppm, versus the monomer (Table 1). A single DP_target_ = 100 was used as it should not significantly influence the gel point as suggested by eq 2 and observed experimentally.^37^ Importantly, to minimize the effect of dilution on the occurrence of intramolecular cyclization,^12,16,35^ the MA/DMSO ratio was kept constant at 1:1 (*v*/*v*), resulting in a relatively high initial monomer concentration [MA]_0_ = 5.55 M. Each crosslinking polymerization was accompanied by an analogous reaction conducted in the absence of HDDA. Both polymerizations were allowed to run until gel was observed in the vial containing the crosslinker.

**Table 1:** Overview of the Parallel Photoinduced ATRP Reactions with Various Catalyst Loadings Conducted in This Work

	[MA]/[EBiB]/[HDDA]	CuBr_2_/Me6TREN (ppm)	with crosslinker		no crosslinker			
entry			a*p*_c,exp_	gelation time (min)	b*M*_n,theo_	b*M*_n,GPC_	cIE	b*D̵*
**1**	100:1:3	200	0.62	25	5890	6970	0.85	1.19
**2**	100:1:3	100	0.55	30	4640	5270	0.88	1.29
**3**	100:1:3	50	0.50	50	4970	6120	0.81	1.30
**4**	100:1:3	20	0.50	70	4520	5040	0.90	1.69
**5**	100:1:3	10	0.48	105	4360	4470	0.98	2.16
**6**	100:1:1.5	200	0.77	35	6830	7540	0.91	1.17
**7**	100:1:1.5	100	0.72	31	6530	7110	0.92	1.20
**8**	100:1:1.5	50	0.70	35	6170	7530	0.82	1.25
**9**	100:1:1.5	20	0.63	70	5320	6180	0.86	1.62
**10**	100:1:1.5	10	0.63	110	5840	6100	0.96	1.86

a Estimated conversion at the gel point, based on data from before gelation and from a corresponding linear polymerization.

b Measured by GPC for a sample taken at a conversion closest to the gel point in a corresponding non-crosslinked reaction. All GPC traces are shown in Figures S1–S10.

c IE = *M*_n,theo_/*M*_n,GPC._

**Figure 1:**
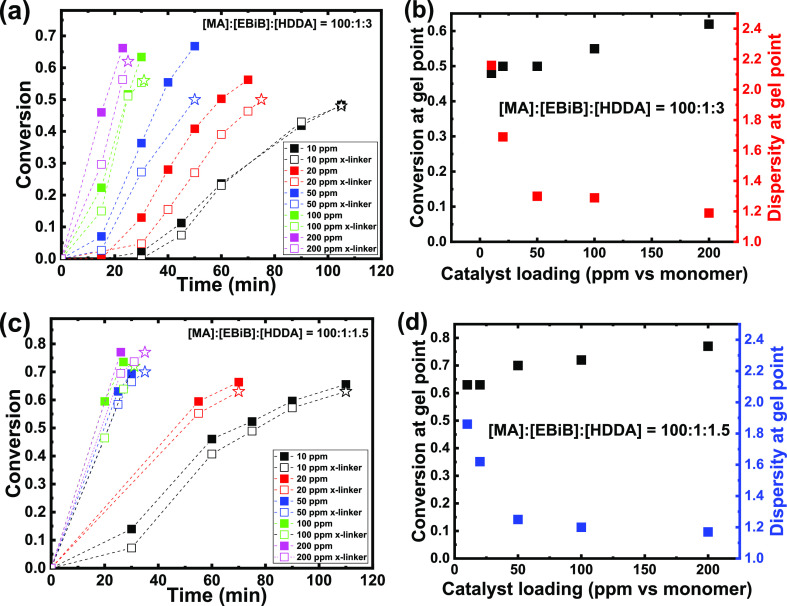
(a,c) Conversion of the vinyl bonds in photoinduced ATRP of MA with (open symbols) and without (closed symbols) the presence of the HDDA crosslinker performed at various CuBr_2_/Me6TREN catalyst loadings. The final open star symbols in polymerizations with HDDA denote the estimated gel point; (b,d) dependence of dispersity and experimental gel points on the catalyst loading. Dispersities were recorded by GPC of the polymerization without a crosslinker at a conversion closest to the experimental gel point. The corresponding GPC traces are shown in Figures S1–S10.

Figure 1a,c shows the conversion versus reaction time plots for all studied conditions. In all cases, the reaction with HDDA was a little slower than its linear counterpart, most likely due to the lower *k*_p_ of HDDA and a slightly higher DP_target_ caused by the addition of a difunctional crosslinker. Decreasing the catalyst loading resulted in slower reactions, which was expected as, in activator regeneration ATRP methods, the overall rate of polymerization (*R*_p_) is controlled by the rate of reduction of Cu^II^ to Cu^I^ which, in turn, depends on the initial concentration of Cu^II^.^41^ GPC traces of all linear polymers show monomodal distributions with MWs close to the theoretical values (Table 1 and Figures S1–S10). The initiation efficiencies (IE_*t*_ = *M*_n,theo_/*M*_n,GPC_) varied between 0.81 and 0.98, and dispersities between 1.17 and 2.16. Notably, the IE_*t*_ values remain constant or are slightly higher for reactions with less catalyst, indicating good control over the polymerization at low catalyst loadings. Indeed, while decreasing the catalyst loading in ATRP with activator regeneration leads to higher *D̵* (due to slower deactivation), it does not result in excessive termination as the radical concentration also decreases with less catalyst (as reflected by slower polymerization).^41^ This is in line with previous works on dispersity tuning in ATRP which showed no loss of control at Cu catalyst loadings down to 10 ppm versus the monomer.^22^

Gelation was monitored by taking samples at time intervals from both reactions and measuring the conversion of the double bonds (MA and HDDA) by ^1^H NMR. Once the system gelled, it was no longer possible to take samples, so the gel point was estimated (open star symbols in Figure 1a,c) based on the previous measurements and the data from the parallel linear polymerization. Due to the abovementioned differences in polymerization rates, gelation took longer with less catalyst; however, it occurred at gradually lower conversions. Specifically, at [EBiB]:[HDDA] ratio = 1:3, conversion at the gel point (*p*_c*,*exp_, Table 1) was 62% when the polymerization was run with 200 ppm of the catalyst (resulting in *D̵* = 1.19) and decreased to 48% at 10 ppm catalyst (*D̵* = 2.16). When the [EBiB]/[HDDA] ratio was lowered to 1:1.5, gel points were naturally higher, but displayed the same trend, that is, gelation occurred at 77% conversion with 200 ppm of the catalyst (*D̵* = 1.17), decreasing to 63% with 10 ppm (*D̵* = 1.86). This trend is in agreement with the previous report of Li et al.^26^ as well as with the FS theory.^4,5^ When less catalyst is used, the rate of deactivation decreases and more monomer/crosslinker units are added to a growing radical during each activation/deactivation cycle, leading to the formation of more high-MW components and thus broadening the MW distribution. These high MW chains will proportionally contain more crosslinks, resulting in earlier gelation than in a homogeneous (i.e., monodisperse) system (see Scheme 1).^5^

### Theoretical Gel Points

As mentioned in the Introduction, the discrepancy between theoretical and experimental gel points is caused primarily by the intramolecular cyclization events occurring in any crosslinking polymerization. In RDRP, experimental gel points are typically observed closer to the theoretical values than in FRP; however, the correct determination of the theoretical gel points is not straightforward. FS theory has long been considered insufficient for the prediction of gel points in RDRP, and more accurate values are usually obtained by various computer simulation methods.^12,15,34−37^

**Figure 2:**
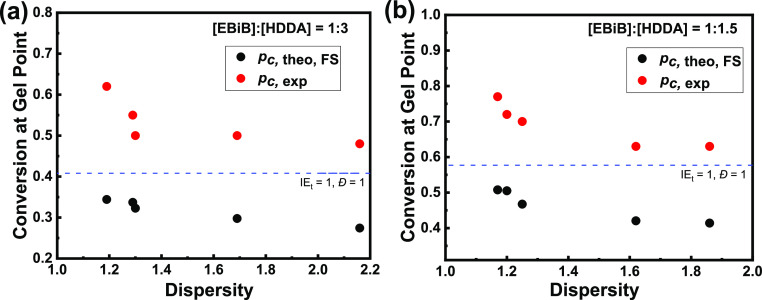
Comparison of experimental (*p*_c*,*exp_) and theoretical FS (*p*_c*,*theo,FS_) gel points calculated from eq 2 using *D̵* and IE_*t*_ values from linear polymerizations. (a) [MA]/[EBiB]/[HDDA] = 100:1:3; (b) [MA]/[EBiB]/[HDDA] = 100:1:1.5. Dashed lines correspond to the ideal case when *D̵* and IE_*t*_ = 1.

Assuming monodisperse chains, eq 2 predicts that the gel point will depend solely on the [*I*]_0_/[*X*]_0_ ratio and reach values of 0.41 and 0.58 for [EBiB]/[HDDA] = 1:3 and 1:1.5, respectively, as indicated by the dashed lines in Figure 2. In order to more precisely calculate gel points for the investigated conditions, we corrected these values using the IE_*t*_ and *D̵* from linear polymerizations (Table 1). This allows us to calculate Flory-Stockmayer theoretical gel points (*p*_c*,*theo,FS_) for each catalyst loading/primary chain dispersity. We note that initiation efficiencies do not vary by more than 0.17, so the effect of *D̵* (ranging from 1.17 to 2.16) is more pronounced.

However, such calculated theoretical gel points differ quite significantly from the experimental values (Figure 2). Previously, Gao et al. used kinetic simulations (Predici) to determine theoretical gel points in ATRP of MA with a crosslinker (assuming ideal living polymerization, i.e., no termination and dispersity given by Poisson distribution *D̵* = 1 + 1/DP_*n*_) and obtained the following empirical expression^12^
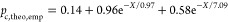
3where *X* = [*X*]_0_/[*I*]_0_. Applying eq 3 to our system gives *p*_c*,*theo,emp_ of 0.58 and 0.82 for [EBiB]/[HDDA] ratios of 1:3 and 1.5, respectively. Both are higher not only than our experimental values but also than the “ideal” cases calculated for *D̵* and IE_*t*_ = 1 from eq 2, highlighting the different scaling of *p*_c,theo_ with [*X*]_0_ in RDRP than that predicted by the FS theory.

Interestingly, for typical values of *X*, eq 3 can be approximated with an excellent agreement by a simple expression  (see Figure S11), suggesting a more general relationship. Indeed, looking at a logarithmic form of eq 2
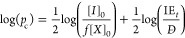
4where *f* is the crosslinker functionality. The second term on the right-hand side of eq 4, namely, , equals 0 when both IE_*t*_ and *D̵* = 1. Indeed, the maximum gel point should occur at *D̵* = 1 (i.e., monodisperse primary chains) and decrease with higher *D̵* (and/or lower IE_*t*_), as predicted by the FS theory and shown experimentally in this work and previously.^26^

The first term, namely, , should therefore give values of *p*_c,theo_ identical to eq 3 for a given [*I*]_0_/[*X*]_0_ ratio, corresponding to a maximum possible gel point in the system, that is, for IE_*t*_ = 1 and *D̵* = 1, similar to those obtained in simulations.^12,15,34−36^ However, this is not the case as all simulated gel points are consistently higher by a factor of  than those calculated from the FS theory (eq 2).

Therefore, we propose a modification of eq 4 to correct for this discrepancy, namely
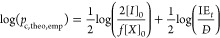
5or
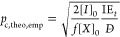
6When *f* = 2, this further reduces to , reflecting the relationship suggested before (Figure S11). Equation 5 or 6 gives the same values as the empirical eq 3 when IE_*t*_ and *D̵* are both equal to unity but allows us to calculate *p*_c*,*theo,emp_ for any IE_*t*_ and *D̵* value.

**Figure 3:**
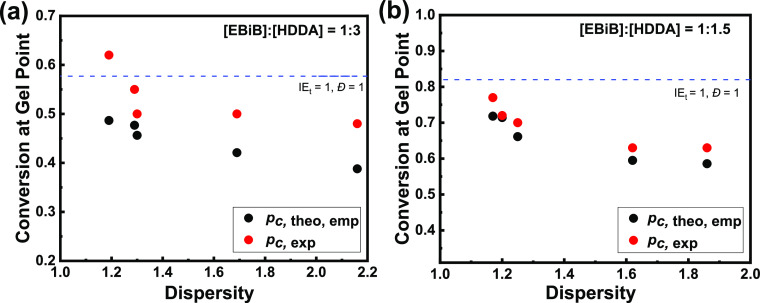
Comparison of experimental (*p*_c,exp_) and theoretical (*p*_c*,*theo,emp_) gel points calculated from eq 6 using *D̵* and IE_*t*_ values from linear polymerizations. (a) [MA]/[EBiB]/[HDDA] = 100:1:3; (b) [MA]/[EBiB]/[HDDA] = 100:1:1.5. Dashed lines correspond to the ideal case when *D̵* and IE_*t*_ = 1.

Inserting our experimental data of IE_*t*_ and *D̵* in eq 6 gives *p*_c*,*theo,emp_ values much closer to the experimental gel points than those calculated previously from eq 2 (Figure 3 and Table 2). However, it should be stressed that while eqs 5 and 6 allow very accurate gel point predictions, in line with previously reported computer simulations, they should be treated as empirical and approximate. Their full derivation would probably require a modification of the FS theory to account for “living” polymerization conditions.^37^ This is beyond the scope of the current study but will be investigated in the future.

**Table 2:** Comparison of Experimental and Theoretical Gel Points Calculated from eqs 2 and 6

entry	[EBiB]_0_/[HDDA]_0_	[cat] (ppm)	IE_*t*_	*D̵*	a*p*_c*,*exp_	b*p*_c,theo,FS_	c*p*_c,theo,emp_
**1**	1:3	200	0.85	1.19	0.62	0.34	0.49
**2**	1:3	100	0.88	1.29	0.55	0.34	0.47
**3**	1:3	50	0.81	1.30	0.50	0.32	0.45
**4**	1:3	20	0.90	1.69	0.50	0.30	0.42
**5**	1:3	10	0.98	2.16	0.48	0.27	0.39
**6**	1:1.5	200	0.91	1.17	0.77	0.51	0.72
**7**	1:1.5	100	0.92	1.20	0.72	0.51	0.71
**8**	1:1.5	50	0.82	1.25	0.70	0.47	0.66
**9**	1:1.5	20	0.86	1.62	0.63	0.42	0.59
**10**	1:1.5	10	0.96	1.86	0.63	0.41	0.59

a Determined experimentally.

b Calculated from eq 2.

c Calculated from eq 6.

Nevertheless, such calculated theoretical gel points still follow the assumptions of the FS theory, namely, equal reactivity of all vinyl bonds and no intramolecular cyclization. As such, they stay below the experimental values, with the average ratio between the theoretical and experimental values *p*_c,theo*,*emp_/*p*_c,exp_ = 0.850 ± 0.056 and 0.948 ± 0.02 for [HDDA]_0_ = 3 and 1.5, respectively, reflecting more intramolecular cyclization at higher crosslinker concentrations. Importantly, this ratio is not influenced by dispersity: even though increasing *D̵* of the primary chains leads to earlier gelation, it should not affect the relative probability of inter- and intramolecular reactions and hence the number of effective crosslinks.

### Mechanical Properties of PMA Networks

**Figure 4:**
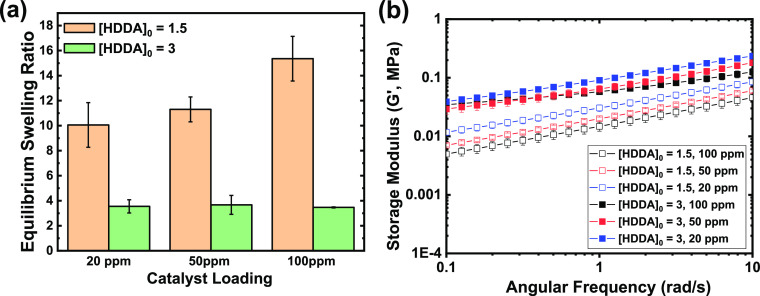
(a) ESRs in acetone and (b) shear storage moduli of dry PMA networks synthesized by photoinduced ATRP with different catalyst loadings. All samples were prepared in triplicate, and shown data are average from at least three separate measurements.

Finally, the impact of changing the catalyst loading/dispersity on the properties of PMA networks was investigated. A series of gels were prepared by continuing the polymerization past the gel point for 10 h to maximize the monomer conversion in all reactions (see Table 3 and Figure S12). Networks were synthesized with 20, 50, and 100 ppm of the catalyst for each crosslinker content (namely, [EBiB]_0_/[HDDA]_0_ = 1:1.5 and 1:3). The obtained network samples were thoroughly washed in acetone and then left in acetone for 24 h to fully swell. The samples were then dried to measure their ESR (Figure 4a). An expected difference in ESR between networks with [HDDA]_0_ = 1.5 and 3 was clearly visible. However, no difference in swelling was observed for networks with [HDDA]_0_ = 3 prepared at various catalyst concentrations as all samples showed ESR ≈ 3.5. Networks with [HDDA]_0_ = 1.5 showed slightly more variation, with ESR ≈ 10, 11, and 15 for 20, 50, and 100 ppm of the catalyst, respectively. This is similar to the recent work by Wanasinghe et al. who reported higher ESR in PMA networks synthesized by PET–RAFT, which displayed lower *D̵* of the primary chains than analogous networks prepared under traditional RAFT conditions and attributed this difference to their more homogeneous structure.^40^ However, given the larger measurement error in networks with [HDDA]_0_ = 1.5, this discrepancy is not substantial.

**Table 3:** ESR and Storage Shear Modulus of PMA Networks Prepared at Different Crosslinker and Catalyst Concentrations

entry	[EBiB]_0_/[HDDA]_0_	[cat] (ppm)	aconversion (%)	bESR	c*G*′ at 0.1 rad s^–1^ (MPa)
**1**	1:3	100	>95	3.5 ± 0.04	0.03
**2**	1:3	50	90	3.7 ± 0.8	0.03
**3**	1:3	20	89	3.6 ± 0.5	0.04
**4**	1:1.5	100	>95	15.4 ± 1.8	0.005
**5**	1:1.5	50	>95	11.3 ± 1.0	0.007
**6**	1:1.5	20	83	10.0 ± 1.4	0.012

a Measured by ^1^H NMR for reactions without HDDA after 10 h.

b Average value from three measurements, determined gravimetrically by swelling in excess acetone for 4 × 24 h.

c Determined by oscillatory rheology for dry networks.

These results were further supported by a rheological analysis of the shear storage moduli (*G*′) of dried networks (Figure 4b). At low angular frequency (0.1 rad/s), corresponding to the relaxed state, virtually no difference in *G*′ of networks with [HDDA]_0_ = 3 and varied dispersities can be seen, corroborating similar crosslinking density suggested by the swelling analysis. Again, more variation was observed in the [HDDA]_0_ = 1.5 series of networks. *G*′ increased with decreasing catalyst loading, from 0.005 MPa for 100 ppm to 0.012 MPa for 20 ppm of the catalyst. However, this difference is relatively small, especially compared with the modulus difference between the samples with different crosslinker contents. Therefore, the effect of changing the dispersity of primary chains on the elastic response of the network is not significant and should only be considered when the material has a low overall crosslinking density. At a higher [*X*]_0_, gelation occurs earlier in the reaction, and the distribution of the crosslinks is more uniform throughout the gel, leading to robust and reproducible networks. Similarly, reducing the catalyst loading also leads to gelation occurring earlier and increases the G′ value of the resulting gel due to the more even crosslink distribution. This effect is apparent in the series of gels with [*X*]_0_ = 1.5. At a higher [*X*]_0_, however, the influence of *D̵* becomes less important as there are enough crosslinks to maintain uniform crosslinking density and produce better-defined networks.

Overall, both swelling analysis and rheology confirm that the dispersity of the primary chains does not greatly affect the properties of polymer networks made by ATRP, which are mainly controlled by the initial [*I*]_0_/[*X*]_0_ ratio. Only at low [*X*]_0_, when gelation occurs at high conversions, increased dispersity of the primary chains can influence the properties by allowing more crosslinks to develop earlier. Furthermore, the occurrence of inevitable intramolecular cyclization, which affects the effective crosslinking density, is not influenced by the primary chain dispersity and is constant for a given [*I*]_0_/[*X*]_0_ ratio, as evidenced by gel point analysis.

## Conclusions

In summary, we investigated how experimental and theoretical gel points are affected by the dispersity of the primary chains in photoinduced ATRP of MA with a diacrylate crosslinker. Decreasing the catalyst loading has two effects on the kinetics of network formation: it results in slower polymerization, that is, longer gelation time; however, it happens at a lower conversion due to the presence of more high MW components and earlier incorporation of the critical number of crosslinks in the primary chains with higher dispersity.

Careful analysis of the theoretical gel points and previously reported kinetic models of gelation in ATRP allowed us to propose a modified FS equation to accurately predict gelation in RDRP techniques. A comparison of theoretical and experimental gel points, combined with the analysis of mechanical properties of the synthesized networks showed that crosslinking density and network structure are not significantly influenced by the MW distribution of the strands and depend primarily on the crosslinker content.

These results can help to better design networks/gels synthesized by ATRP and other RDRP techniques, as well as guide efforts to control or even eliminate the occurrence of intramolecular cyclization reactions in chain-growth polymerization, leading to defect-free polymer networks. From a more practical perspective, Cu catalyst loading in ATRP can be diminished in network synthesis without a significant impact on their mechanical properties, which can be useful, for example, in the preparation of hydrogels for biomedical applications.

## Supporting Information Available

Derivation of eq 2 and GPC traces (PDF)
