# Supplementary material for: Gelation in Photoinduced ATRP with Tuned Dispersity of the Primary Chains
Source: Macromolecules. 2023 Feb 22;56(5):2009–16. doi: 10.1021/acs.macromol.2c02159 (PMC10018774; doi:10.1021/acs.macromol.2c02159)
Supplement: Supplementary file 1 — ma2c02159_si_001.pdf [file ma2c02159_si_001.pdf]

**Supporting Information**

# Gelation in Photoinduced ATRP with Tuned Dispersity of the Primary Chains

Frances Dawson, Hugo Jafari, Vytenis Rimkevicius, Maciej Kopeć*

Department of Chemistry, University of Bath, Claverton Down, Bath BA2 7AY, UK

*e-mail: mk2297@bath.ac.uk

**Flory-Stockmayer equation for ATRP systems**

Derivation of the equation 2 after Gao and Matyjaszewski[1,2] is reproduced here for reader's convenience.

From the Flory-Stockmayer theory:

$$v_c = \rho p_c (DP_w - 1) = 1$$

where is $v_c$ is the weight-average number of crosslinks per primary chain (equal to 1 at incipient gelation), $\rho$ is the fraction of vinyl bonds residing on the divinyl crosslinker, $p_c$ is the conversion of double bonds at the gel point and $DP_w$ is the weight-average degree of polymerisation of the primary chains in the absence of crosslinks.

Assuming $DP_w >> 1$ and since $Đ = DP_w/DP_n$ as well as $\rho = \frac{2[X]_0}{[M]_0 + 2[X]_0}$

then,

$$1 = \frac{2[X]_0}{[M]_0 + 2[X]_0} p_c Đ DP_n$$

Further, in an ATRP of a monomer with initial concentration $[M]_0$ and a divinyl crosslinker with initial concentration of vinyl bonds $2[X]_0$, $DP_n = \frac{[M]_0 + 2[X]_0}{[PC]_t} p_c$, where $[PC]_t$ is the instantaneous concentration of the primary chains.

Thus,

$$1 = \frac{2[X]_0}{[M]_0 + 2[X]_0} p_c Đ \frac{[M]_0 + 2[X]_0}{[PC]_t} p_c = p_c^2 Đ \frac{2[X]_0}{[PC]_t}$$

Finally, conversion at the gel point:

$$p_c = \sqrt{\frac{[PC]_t}{2[X]_0} \frac{1}{Đ}}$$

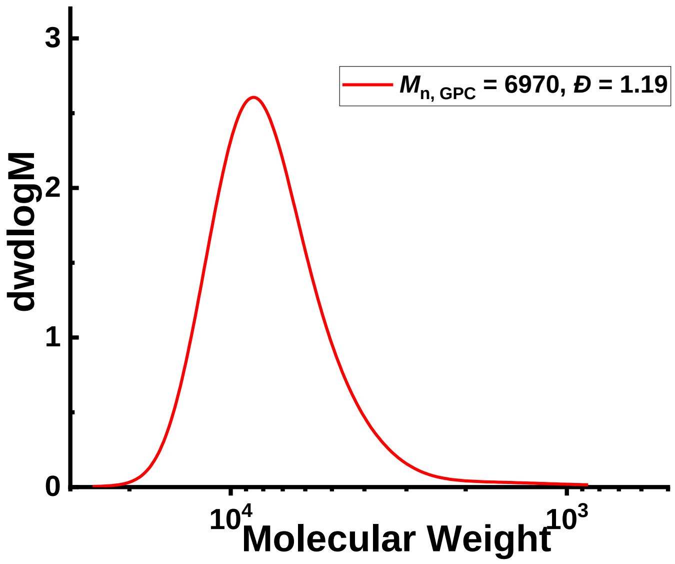


**Figure S1**. GPC trace of a reaction with conditions [MA]:[EBiB] = 100:1 and 200 ppm of the $CuBr_2$/ $Me_6TREN$ catalyst (**Table 1, entry 1**). The sample was taken after 23 min and the polymerization reached 66% conversion measured by $^1$H NMR. The parallel reaction with 3 equiv, of the HDDA crosslinker gelled in 25 min and the conversion at the gel point was estimated to be 62%.

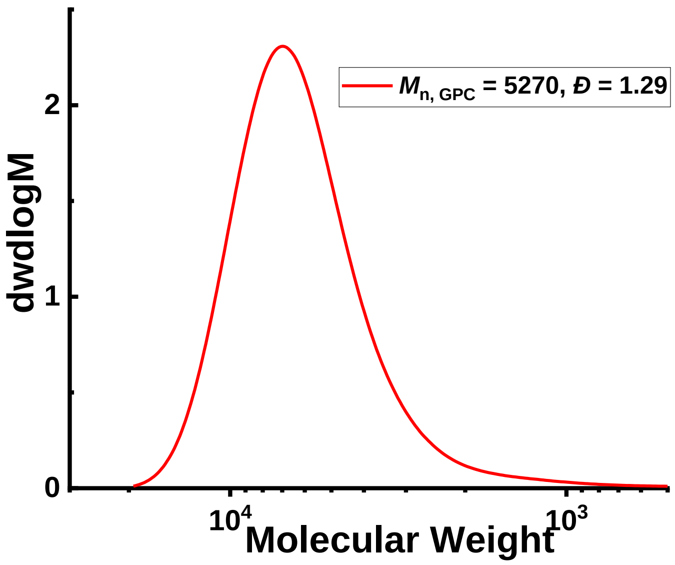


**Figure S2**. GPC trace of a reaction with conditions MA/EBiB 100:1 and 100 ppm of the $CuBr_2$/ $Me_6TREN$ catalyst (**Table 1, entry 2**). The sample was taken after 25 min and the polymerization reached 52% conversion measured by $^1$H NMR. The parallel reaction with 3 equiv, of the HDDA crosslinker gelled in 30 min and the conversion at the gel point was estimated to be 55%.

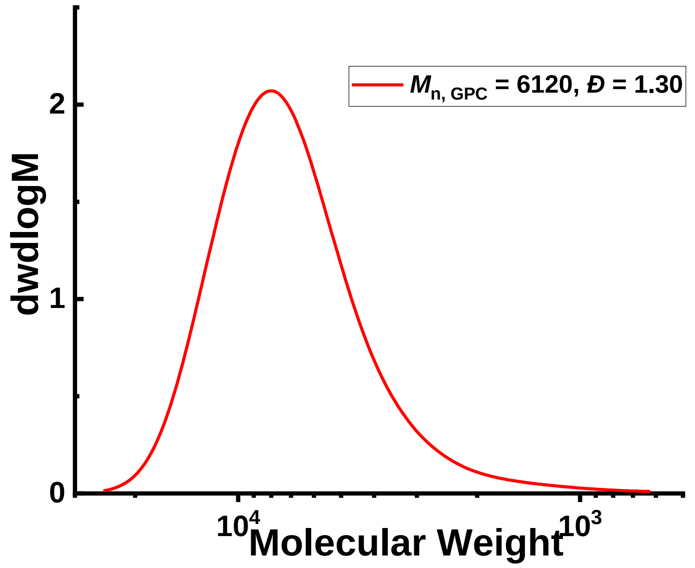


**Figure S3**. GPC trace of a reaction with conditions [MA]:[EBiB] = 100:1 and 50 ppm of the $CuBr_2$/ $Me_6TREN$ catalyst (**Table 1, entry 3**). The sample was taken after 40 min and the polymerization reached 55% conversion measured by $^1$H NMR. The parallel reaction with 3 equiv, of the HDDA crosslinker gelled in 25 min and the conversion at the gel point was estimated to be 50%.

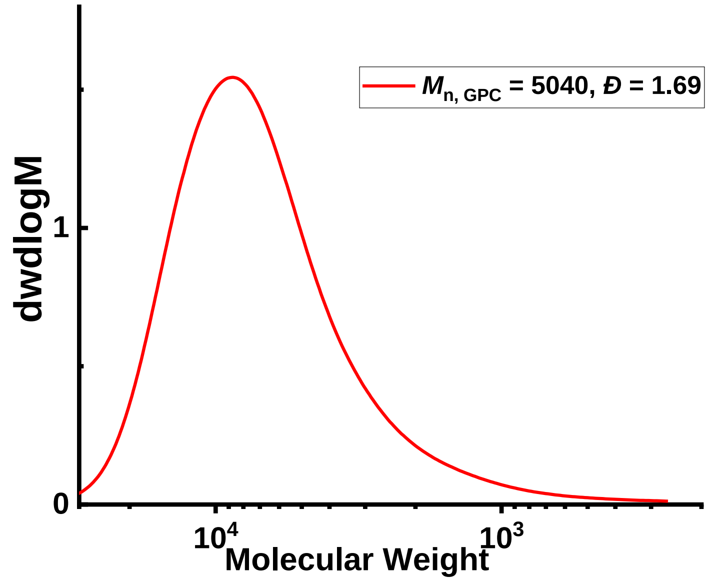


**Figure S4**. GPC trace of a reaction with conditions [MA]:[EBiB] = 100:1 and 20 ppm of the $CuBr_2$/ $Me_6TREN$ catalyst (**Table 1, entry 4**). The sample was taken after 60 min and the polymerization reached 50% conversion measured by $^1$H NMR. The parallel reaction with 3 equiv, of the HDDA crosslinker gelled in 70 min and the conversion at the gel point was estimated to be 50%.

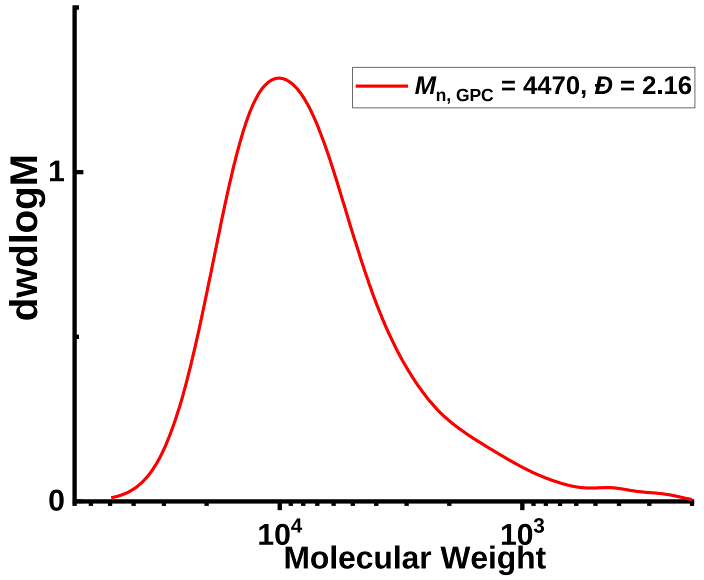


**Figure S5**. GPC trace of a reaction with conditions [MA]:[EBiB] = 100:1 and 10 ppm of the $CuBr_2$/ $Me_6TREN$ catalyst (**Table 1, entry 5**). The sample was taken after 105 min and the polymerization reached 48% conversion measured by $^1H$ NMR. The parallel reaction with 3 equiv, of the HDDA crosslinker gelled in 105 min and the conversion at the gel point was estimated to be 48%.

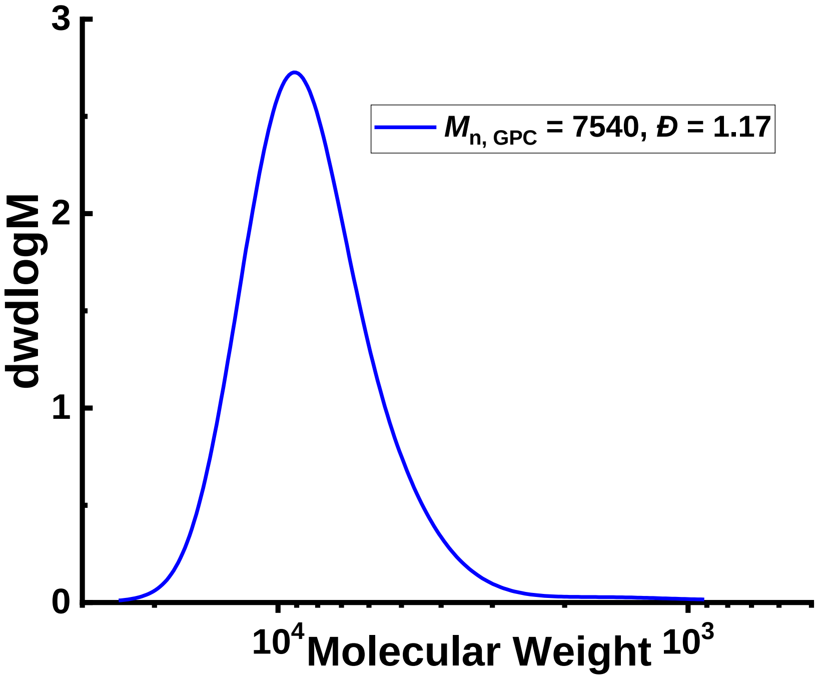


**Figure S6**. GPC trace of a reaction with conditions [MA]:[EBiB] = 100:1 and 200 ppm of the $CuBr_2$/ $Me_6TREN$ catalyst (**Table 1, entry 6**). The sample was taken after 26 min and the polymerization reached 77% conversion measured by $^1H$ NMR. The parallel reaction with 1.5 equiv, of the HDDA crosslinker gelled in 35 min and the conversion at the gel point was estimated to be 77%.

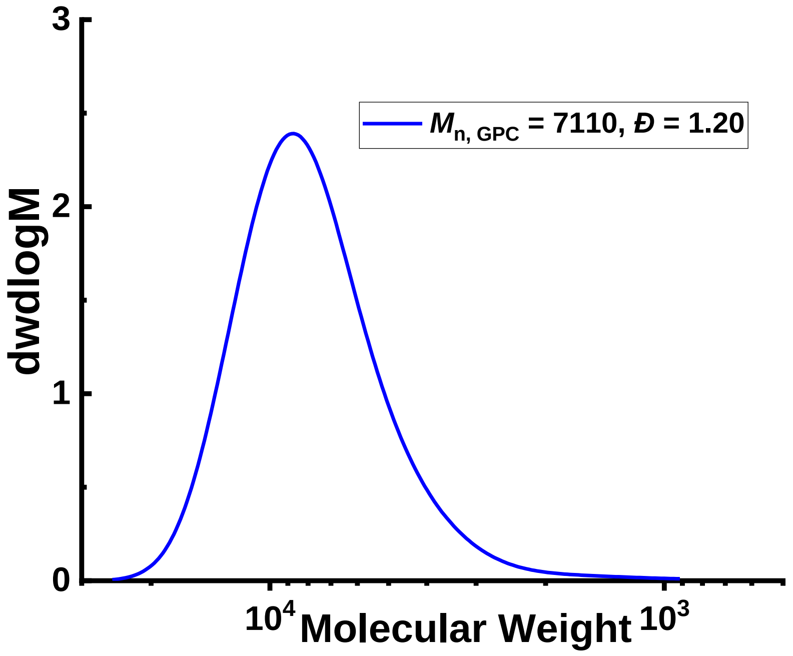


**Figure S7**. GPC trace of a reaction with conditions [MA]:[EBiB] = 100:1 and 100 ppm of the $CuBr_2$/ $Me_6TREN$ catalyst (**Table 1, entry 7**). The sample was taken after 27 min and the polymerization reached 74% conversion measured by $^1H$ NMR. The parallel reaction with 1.5 equiv, of the HDDA crosslinker gelled in 31 min and the conversion at the gel point was estimated to be 72%.

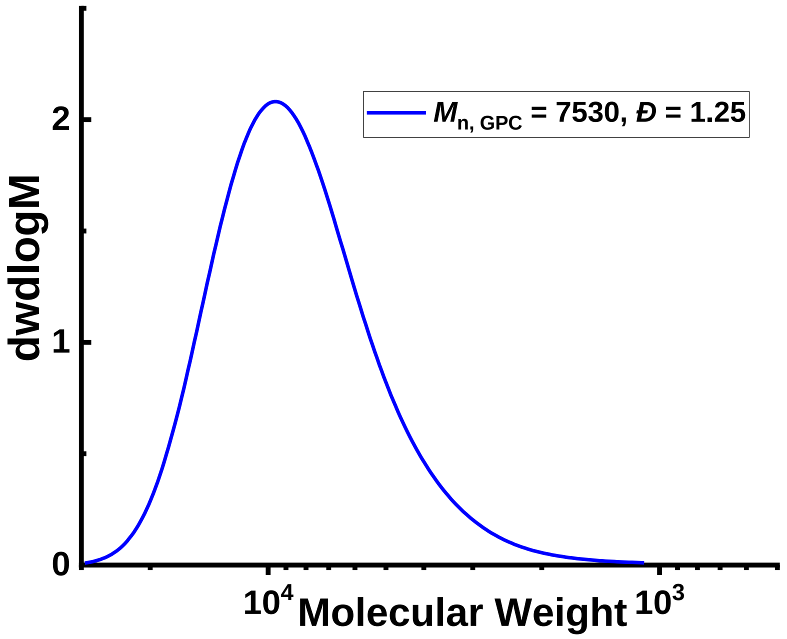


**Figure S8**. GPC trace of a reaction with conditions [MA]:[EBiB] = 100:1 and 50 ppm of the $CuBr_2$/ $Me_6TREN$ catalyst (**Table 1, entry 8**). The sample was taken after 30 min and the polymerization reached 69% conversion measured by $^1H$ NMR. The parallel reaction with 1.5 equiv, of the HDDA crosslinker gelled in 35 min and the conversion at the gel point was estimated to be 70%.

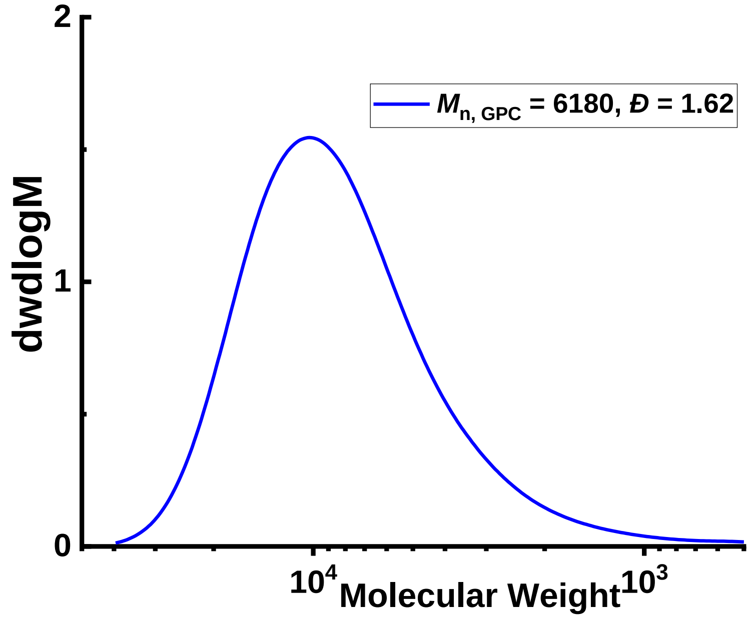


**Figure S9**. GPC trace of a reaction with conditions [MA]:[EBiB] = 100:1 and 20 ppm of the $CuBr_2$/ $Me_6$TREN catalyst (**Table 1, entry 9**). The sample was taken after 55 min and the polymerization reached 60% conversion measured by $^1$H NMR. The parallel reaction with 1.5 equiv, of the HDDA crosslinker gelled in 70 min and the conversion at the gel point was estimated to be 63%.

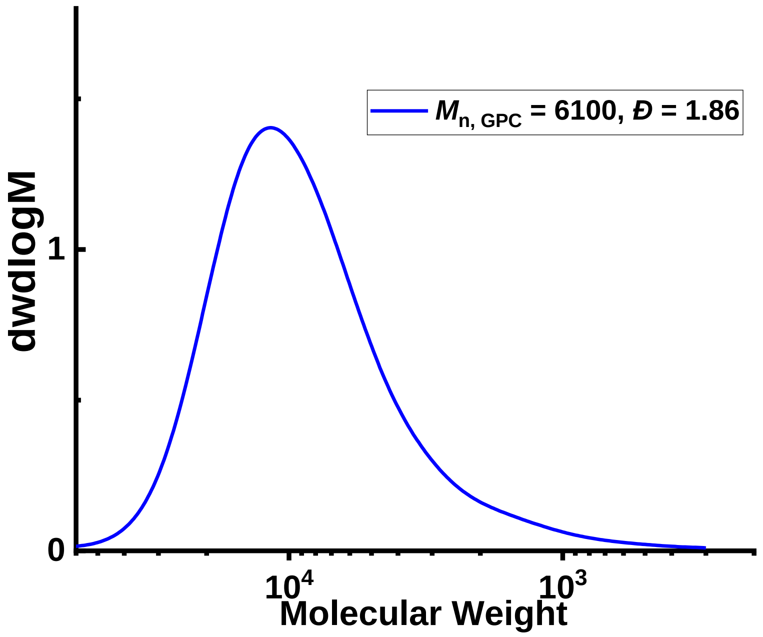


**Figure S10**. GPC trace of a reaction with conditions [MA]:[EBiB] = 100:1 and 10 ppm of the $CuBr_2$/ $Me_6$TREN catalyst (**Table 1, entry 10**). The sample was taken after 110 min and the polymerization reached 66% conversion measured by $^1$H NMR. The parallel reaction with 1.5 equiv, of the HDDA crosslinker gelled in 110 min and the conversion at the gel point was estimated to be 63%.

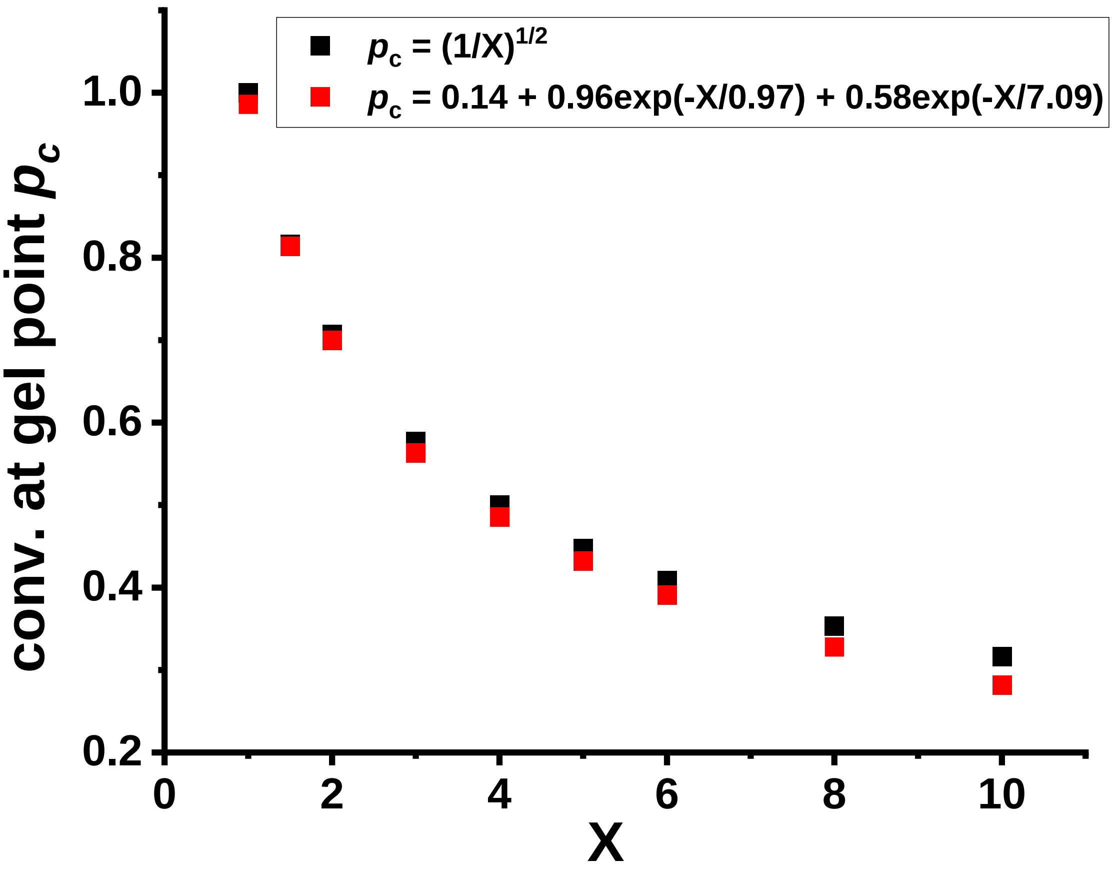


**Figure S11**. Comparison of two formulas to calculate $p_c$ as a function of the initial crosslinker concentration $X = [X]_0/[I]_0$. The second order exponential decay function (red squares) was obtained in ref. 3 using Predici simulation software.

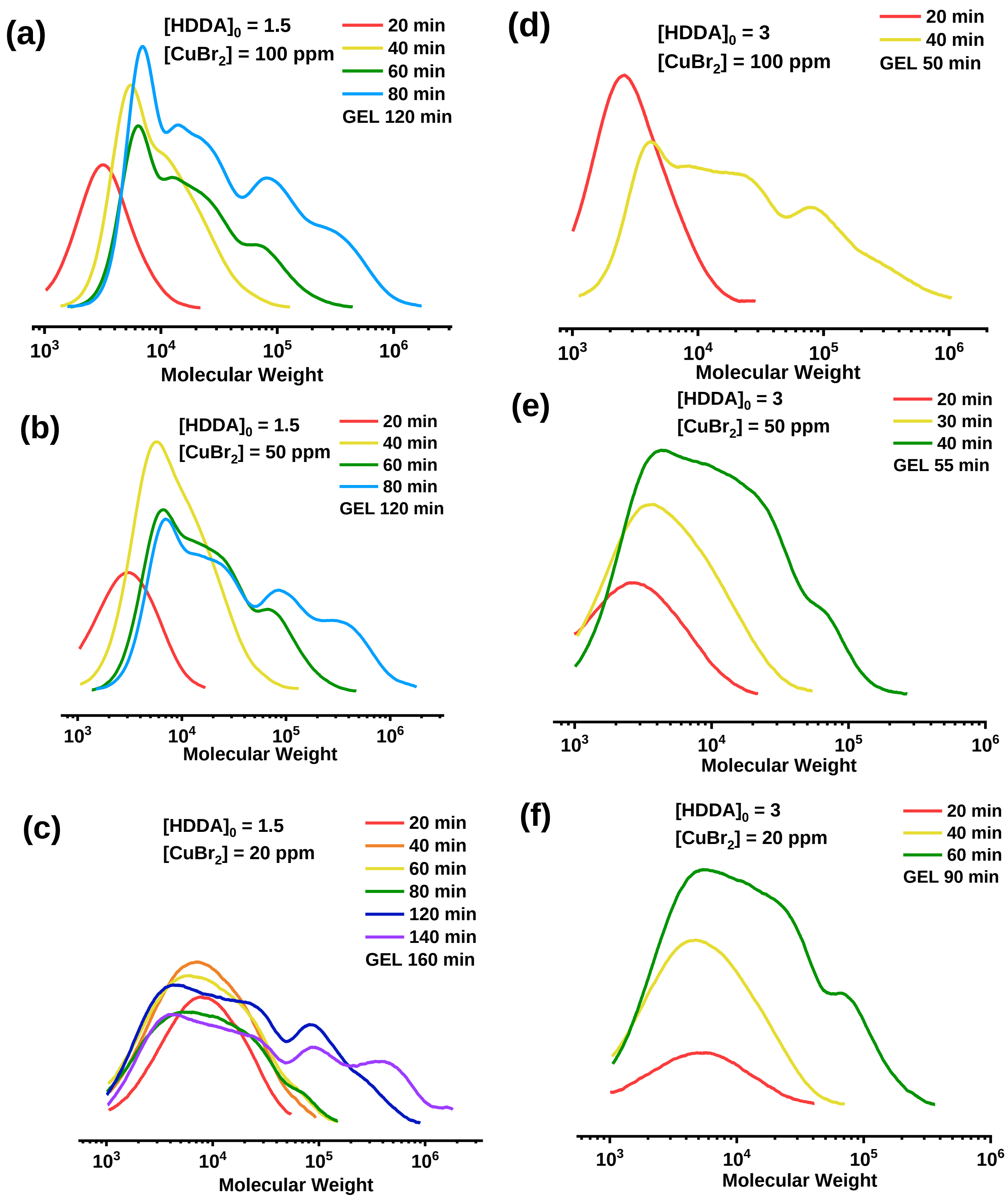


**Figure S12.** GPC traces recorded pre-gelation for reactions with 20-100 ppm of the catalyst used to synthesize fully developed networks for structural characterization. (a-c) $[HDDA]_0$ = 1.5; (e-g) $[HDDA]_0$ = 3. Reaction conditions: [MA]:[EBiB]:[HDDA] = 100:1:1.5 or 3; $[CuBr_2]$ = 20-100 ppm vs monomer; $[CuBr_2]$:[Me6TREN] = 1:6, MA/DMSO = 1:1 (v/v), rt, $\lambda_{max}$ = 390 nm, 10 h. Note that for 10-hour reactions, the LED lamp (Kessil PR106L-390) was set to 50% intensity, hence longer reaction/gelation times.

**References**


1. Gao, H.; Matyjaszewski, K., Synthesis of functional polymers with controlled architecture by CRP of monomers in the presence of cross-linkers: From stars to gels. *Prog. Polym. Sci.* **2009,** *34* (4), 317-350.
2. Gao, H.; Li, W.; Min, K.; Matyjaszewski, K., Gelation in Atom Transfer Radical Copolymerization with a Divinyl Cross-linker. In *Controlled/Living Radical Polymerization: Progress in ATRP*, American Chemical Society: 2009; Vol. 1023, pp 203-213.
3. Gao, H.; Min, K.; Matyjaszewski, K., Determination of Gel Point during Atom Transfer Radical Copolymerization with Cross-Linker. *Macromolecules* **2007,** *40* (22), 7763-7770.
